# Non-operative management of blunt hepatic and splenic injuries–practical aspects and value of radiological scoring systems

**DOI:** 10.1007/s10353-018-0545-x

**Published:** 2018-07-20

**Authors:** Margot Fodor, Florian Primavesi, Dagmar Morell-Hofert, Matthias Haselbacher, Eva Braunwarth, Benno Cardini, Eva Gassner, Dietmar Öfner, Stefan Stättner

**Affiliations:** 1 Department of Visceral, Transplantation and Thoracic Surgery, Medical University of Innsbruck, Anichstraße 35, 6020 Innsbruck, Austria; 2 Department of Radiology, Medical University of Innsbruck, Anichstraße 35, 6020 Innsbruck, Austria; 3 Department of Trauma Surgery, Medical University of Innsbruck, Anichstraße 35, 6020 Innsbruck, Austria

**Keywords:** Abdominal trauma, Sports, Multiple trauma, Classification, Diagnostic imaging, Bauchtrauma, Sport, Multiple Traumata, Klassifikation, Diagnostische Bildgebung

## Abstract

**Background:**

Non-operative management (NOM) of blunt hepatic and splenic injuries has become popular in haemodynamically stable adult patients, despite uncertainty about efficacy, patient selection, and details of management. Up-to-date strategies and practical recommendations are presented.

**Methods:**

A selective literature search was conducted in PubMed and the Cochrane Library (1989–2016).

**Results:**

No randomized clinical trial was found. Non-randomized controlled trials and large retrospective and prospective series dominate. Few systematic reviews and meta-analyses are available. NOM of selected patients with blunt liver and spleen injuries is associated with low morbidity and mortality. Only data of limited evidence are available on intensity and duration of patient monitoring, repeat imaging, antithrombotic prophylaxis and return to normal activity. There is high-level evidence on early mobilisation and post-splenectomy vaccination.

**Conclusion:**

NOM of blunt liver or spleen injuries is a worldwide trend, but the literature does not provide high-grade evidence for this strategy.

## Zusammenfassung

**Hintergrund:**

Das nichtoperative Management (NOM) stumpfer Leber- und Milzverletzungen hat sich zu einem beliebten Verfahren bei hämodynamisch stabilen Patienten entwickelt, trotz der Ungewissheit bezüglich Wirksamkeit, Patientenselektion und Details zum Management. In diesem Beitrag werden aktuelle Verfahren und praktische Empfehlungen vorgestellt.

**Methoden:**

Eine selektive Literaturrecherche in PubMed und Cochrane Library (1989–2016) wurde durchgeführt.

**Ergebnisse:**

Es wurde keine randomisierte klinische Studie gefunden. Nichtrandomisierte kontrollierte Studien und große retrospektive und prospektive Serien dominieren das Ergebnis. Einzelne systematische Reviews und Metaanalysen sind verfügbar. Das NOM bei ausgewählten Patienten mit stumpfen Leber- und Milzverletzungen ist mit einer geringen Morbidität und Mortalität assoziiert. Hinsichtlich der Intensität und Dauer der Patientenüberwachung, der wiederholten Bildgebung, Thromboseprophylaxe und Wiederaufnahme der normalen Aktivitäten stehen nur Daten mit eingeschränkter Evidenz zur Verfügung. Es besteht eine hochgradige Evidenz für die frühe Mobilisation und die Immunisierung nach Splenektomie.

**Schlussfolgerung:**

Das NOM stumpfer Leber- und Milzverletzungen ist ein weltweiter Trend, jedoch bietet die Literatur keine hochgradige Evidenz für dieses Verfahren.

## Main novel aspects

- This continuing education article describes the current state of the art of NOM of blunt hepatic and splenic injuries.
- Practical clinical recommendations are presented.
- Proposed injury scales are reviewed.

## Learning objectives

After reading this article, you should …

- understand the rational for NOM blunt hepatic and splenic injuries.
- be able to apply the criteria for appropriate patient selection.
- be familiar with the strengths and weaknesses of current classification and scoring systems for injuries of the liver and spleen.
- be able to critically discuss the role of CT in guiding hepatic and splenic injury management.
- know the therapeutic strategies for NOM, including details of monitoring and follow-up.
- understand the importance of post-splenectomy and post-embolisation vaccination.

## Introduction

Blunt abdominal trauma is very common in central European emergency departments, especially due to sports activities. Management of these injuries can be challenging because of the frequent association with a complex clinical picture of abdominal, thoracic, limb and head trauma [1, 2].

## Background

### Therapeutic strategies

Abdominal organs are involved in 30% of **polytrauma**

polytrauma

patients, with an occurrence of hepatic and splenic injuries in 13 and 16%, respectively [3]. Therapeutic strategies contain either non-operative management (NOM) including radiological interventions (**angiography**

angiography),

or surgical treatment. Due to frequent postoperative complications after primary surgical treatment in the past,

Haemodynamically stable patients are nowadays frequently managed non-operatively

a paradigm shift to NOM in haemodynamically stable patients has emerged in major trauma centres [3, 4], where NOM has been described as a safe procedure when twenty-four-seven availability of experienced surgeons, modern imaging modalities, intensive care units (ICU) and other support services [5] is assured.

This shift was largely driven by intraoperative observations that found many minor liver and splenic injuries to no longer be bleeding [2, 6, 7],

Many minor hepatic und splenic injuries were intraoperatively found to require no further surgical intervention

with no further surgical intervention necessary. NOM is associated with lower hospital costs, fewer non-therapeutic **laparotomies**

laparotomies,

a lower rate of intra-abdominal complications, lower rates of blood transfusion, and decreased morbidity and mortality [8, 9].

### Patient selection criteria

The decision to employ a NOM pathway for blunt hepatic and splenic injuries requires the patient to meet several criteria, primarily that of **haemodynamic stability**

haemodynamic stability.

Although many of the developed algorithms and institutional protocols addressing NOM of splenic injuries include contraindications such as advanced patient age, higher splenic injury grades and distinct transfusion triggers, the lion’s share of these factors are more individualized algorithms than strict, scientifically evidenced conventions. The recent 2012 edition of the Eastern Association for the Surgery of Trauma (EAST) guidelines for the management of blunt hepatic injuries are comparable to those for blunt splenic injuries, with the possibility of adjunct interventions including angiography, endoscopic retrograde **cholangiopancreatography**

cholangiopancreatography

(ERCP) and laparoscopy [10].

Haemodynamically instable patients or those with peritonitis warrant operative intervention

Haemodynamically unstable patients or those affected by peritonitis still warrant operative intervention. However, many questions regarding practical aspects of NOM are still without conclusive answers in the guidelines, despite the large amount of recent literature on this topic. For example, there are few clear recommendations regarding the following aspects: frequency of clinical examination, imaging procedures and **haemoglobin**

haemoglobin

measurements; intensity and duration of **monitoring**

monitoring;

transfusion triggers after which operative or angiographic intervention should be considered; time to re-initiating oral intake; duration and intensity of restricted activity during hospitalisation and after discharge; optimum length of stay (LOS) within the ICU and hospital; timing of initiating venous **thromboembolic prophylaxis**

thromboembolic prophylaxis;

the role of post-splenic injury vaccines and post-discharge follow-up [11].

## Objective

This summary focuses on current state-of-the-art practical management recommendations, including a review of proposed injury scales. Grades of evidence are based on the approvals of the Oxford Centre for Evidence-Based Medicine [12, 13].

## Classification of liver and splenic trauma

An accurate classification system forms the basis for the clinical decision-making process.

Advanced imaging techniques enable new radiological classification systems

Advances in imaging techniques have led to the development of newer radiological classification systems; however, the accuracy of these remains to be validated [14]. Despite their widespread acceptance and use, these gradings have been subjected to limited independent studies, and only a few of the scales have been validated for outcomes on a large-scale basis [15].

## Trauma scoring systems

Trauma scores were introduced more than 30 years ago for assigning numerical values to anatomic lesions and physiological changes after an injury. The purpose of a 

Scaling systems measure lesion severity and facilitate clinical decisions

scaling system for specific injuries is to measure lesion severity and provide a common language to facilitate clinical decisions [16]. In view of the complex clinical picture and variety of treatment options, any modern classification scheme for trauma should be able to fulfil a series of general principles:

- First of all, it should be able to identify the mechanism of trauma, as well as the extent and anatomic details of the injuries.
- Second, it should be able to recognize distinctive groups of injuries, which could be correlated with a certain management strategy, either conservative or surgical.
- Third, the grades of trauma should be linked with the likelihood of further complications and potential outcome.
- Finally, any classification has to be objective and reproducible, and must have prospective as well as retrospective applicability [14].

### AAST Score

The most accepted scoring system is the “Moore score” (AAST Score), based on the Organ Injury Scale (OIS) of the American Association for Surgery of Trauma (AAST) published in 1989 [17].

The Moore score is a gold standard for spleen and liver injuries

The Moore score is considered a gold standard to describe spleen and liver injuries [18]. This classification scheme is an anatomic description scaled from I to V for the spleen and from I to VI for the liver, representing minimal to the most severe injury (Tables 1 and 2; [12, 17]). Most liver injuries are grade I, II or III, and are successfully treated conservatively. In contrast, the majority of grade IV, V or VI liver injuries necessitate operative intervention.

Success rates of NOM of hepatic trauma range from 82 to 100%

The success rate of NOM of hepatic trauma ranges from 82 to 100% [19, 20]. Similarly, in splenic injury, low-AAST-grade lesions (I–III) are frequently treated with NOM, facilitating good results in terms of morbidity and mortality [13]. However, many patients with

Even high-grade hepatic and splenic lesions in haemodynamically stable patients can be treated successfully with NOM

even high-grade lesions of the liver or spleen may be haemodynamically stable and treated successfully with NOM [13, 19] in specialised centres. Hence, a contemporary NOM algorithm for splenic and hepatic injuries should also consider the haemodynamic status and other associated injuries, e. g. through incorporation of the Injury Severity Score (ISS).

**Table 1:** Moore classification/AAST spleen injury scale (1994 revision)

Grade^a^	Type	Injury description
I	Haematoma	Subcapsular, <10% surface area
	Laceration	Capsular tear, <1% parenchymal depth
II	Haematoma	Subcapsular, 10–50% surface area, intra-parenchymal <5 cm in diameter
	Laceration	Capsular tear, 1–3 cm parenchymal depth that does not involve a trabecular vessel
III	Haematoma	Subcapsular, >50% surface area or expanding; ruptured subcapsular or parenchymal haematoma, intra-parenchymal haematoma ≥5 cm or expanding
	Laceration	>3 cm parenchymal depth or involving trabecular vessels
IV	Laceration	Laceration involving segmental or hilar vessels producing major devascularisation (>25% of spleen)
V	Laceration	Complete shattered spleen
	Vascular	Hilar vascular injury which devascularises spleen

*AAST* American Association for Surgery of Trauma

^a^Advance one grade for multiple injuries, up to grade III

**Table 2:** Moore classification/AAST liver injury scale (1994 revision)

Grade^a^	Type	Injury description
I	Haematoma	Subcapsular, <10% surface area
	Laceration	Capsular tear, <1 cm parenchymal depth
II	Haematoma	Subcapsular, 10–50% surface area, intra-parenchymal <10 cm in diameter
	Laceration	1–3 cm parenchymal depth, <10 cm in length
III	Haematoma	Subcapsular, >50% surface area or expanding; ruptured subcapsular or parenchymal haematoma, intra-parenchymal haematoma ≥10 cm or expanding
	Laceration	>3 cm parenchymal depth
IV	Laceration	Parenchymal disruption involving 25–75% of hepatic lobe or 1–3 Couinaud’s segments within the single lobe
V	Laceration	Parenchymal disruption involving >75% of hepatic lobe or >3 Couinaud’s segments within the single lobe
	Vascular	Juxtavenous hepatic injuries, i. e. retrohepatic vena cava/central major hepatic veins
VI	Vascular	Hepatic avulsion

*AAST* American Association for Surgery of Trauma

^a^Advance one grade for multiple injuries, up to grade III

The primary objective of the AAST classification was to provide a common language to describe specific organ injuries and facilitate clinical decision-making. The OIS committee of AAST was organized in 1987 with the purpose of developing injury severity scales for individual organs. The first OIS grading systems for spleen, liver and kidney were published in 1988. A study performed by Tinkoff et al. [15] attempted to validate the OIS score for spleen, liver and kidney injuries using the National Trauma Data Bank. Injuries were stratified by each organ either as an isolated injury or in combination with other intra-abdominal or extra-abdominal injuries. For all groups, each OIS grade was then analysed for mortality, hospital LOS, duration of ICU stay and hospital costs. For patients with combined abdominal injuries, higher OIS rates were associated with an increase in mortality, organ-specific operative rate and hospital costs. Also, when isolated organ injuries were examined, increasing OIS grade significantly correlated with these outcome variables [15].

The introduction and

Modern CT techniques have had a huge impact on the classification and management of splenic and hepatic trauma

refinement of CT scanning has had a great impact on the classification and subsequent management of spleen and liver trauma. CT scanning is able to identify sub-capsular or central **haematomas**

haematomas,

**contusions**

contusions,

**periportal tracking**

peri-portal tracking

of fluid as well as complex **lacerations**

lacerations

and **fragmentations**

fragmentations.

### Other proposed CT-based classifications

Despite the AAST classification incorporating extensive preoperative assessment data derived from the CT examination, additional CT-based classification schemes were developed. Mirvis et al. proposed a five-grade CT-based scheme of hepatic injury varying from capsular **avulsion**

avulsion

to major parenchymal damage and vascular injury [8]. However, the current scoring systems do not incorporate the localization of injury and the mechanism of trauma [18]. Hence, the role of CT grading of blunt abdominal injuries is still controversial. Some authors investigated whether CT findings can determine whether patients require surgery or can be managed conservatively. Retrospective studies evaluated CT grading of splenic injuries and supported the hypothesis that properly selected patients can be safely observed, regardless of the magnitude of splenic injury on CT scans.

The decision for early surgical exploration should be based on clinical criteria

A decision to undergo early surgical exploration should be based on clinical criteria, including the patient’s age and associated injuries [21]. This is also underlined by findings of Sutyak et al., noticing that CT may be inaccurate for estimating the severity of adult splenic injury. This group compared CT results with operative findings and analysed outcome of NOM as well as CT reproducibility accordingly. The CT score correctly predicted intraoperative grade in 10 patients, underestimated it in 18 and overestimated it in 6. Splenic injury was missed in 5 patients. Radiologists disagreed on 20% of the scans. In conclusion, splenic injury management should not be based solely on CT severity and on CT-based scoring systems [22]. In another study, abdominal CT scans obtained on admission were retrospectively reviewed over a 3-year period. CT-based injury scores were compared with clinical outcome in patients treated either surgically or conservatively. The results indicated that even major hepatic injury assessed with preoperative CT can usually be managed without surgery in haemodynamically stable patients [18].

In summary, an accurate clinical classification of hepatic and splenic injuries correctly determining the appropriate treatment strategy as well as predicting subsequent outcome is still pending [14]. In the majority of cases, scoring is only related to the degree of injury to a particular organ, while the outcome depends not only on injury to a single organ, but rather on the totality of all injuries and the patient’s existing morbidities.

Therefore, most centres will rely on algorithms combining different parameters such as haemodynamic stability, imaging findings and timely availability of interventional techniques to decide whether to opt for immediate surgery or attempt NOM.

## Success and failure of non-operative management

Contradictory findings are reported concerning **prognostic factors**

prognostic factors

for failure of NOM in blunt abdominal trauma. In a survey performed by Olthof et al., the **Delphi method**

Delphi method

was used to reach consensus among 30 expert trauma surgeons and **interventional radiologists**

interventional radiologists

for the NOM of patients with blunt splenic injuries. An agreement of 80% or greater was defined as consensus. Results indicated that awareness for unsuccessful NOM is required in patients aged 40 years or older, in patients with an ISS of 25 or higher, or those with splenic injury grade III or higher [23].

Blunt trauma patients with concomitant injury to liver and spleen have higher ISS and an increase in mortality, LOS and transfusion requirements. Hence, the risk of failure with NOM is increased and

Extra vigilance is warranted during non-operative management of patients with trauma to both liver and spleen

extra vigilance is therefore warranted when choosing this form of therapy in the presence of injury to both organs [24]. In a retrospective protocol-driven study performed by Hsieh et al., a failure rate of 4.9% was described [25]. Other parameters that might be helpful in predicting the failure of NOM are **lactate**

lactate

levels on admission, necessity of transfusion, **crystalloid resuscitation**

crystalloid resuscitation

and drop-in **haematocrit**

haematocrit

levels during the first hour after admission [26].

Strict protocols for follow-up of patients treated with NOM are essential. According to Peitzman et al., only one third of trauma centres have well-established NOM protocols for spleen injuries [27]. Only 20% of experts from the AAST consider that the protocol established in their institution is well supported by the available literature [28].

## Physical and clinical examination

The value of physical examination is often underestimated, but increased temperature or respiratory rate can indicate an intra-abdominal perforation or the presence of an **abscess**

abscess.

Pulse and blood pressure can also change with **sepsis**

sepsis,

intra-abdominal bleeding or **peritonitis**

peritonitis.

Thorough physical examination is very important for determining the necessity of exploratory laparotomy

A carefully performed physical examination remains one of the most important methods to determine the need for exploratory laparotomy [29]. The initial clinical assessment is often difficult and inaccurate. Tenderness and spasm caused by associated injuries in the abdominal wall may make diagnosis challenging. Abdominal wall injury contributes to clinical error in diagnosing injury of the intra-abdominal viscera but more importantly, free peritoneal blood may cause peritoneal irritation. Several studies have highlighted the issues related to physical examination in blunt abdominal trauma. In a retrospective analysis performed by Schurink et al., physical examination was shown to be possibly unreliable in assessing abdominal organ involvement in patients with multiple injuries or isolated head injuries [30]. Also, while most conscious patients with severe intra-abdominal injuries will usually present with either abdominal pain or tenderness, there is a small group of awake and alert patients in whom the physical examination will be falsely negative due to associated extra-abdominal injuries [31]. However,

Repeated clinical examination combined with modern imaging and laboratory investigations plays a key role in clinical decision-making

repeated clinical examination supplemented with modern imaging and laboratory investigations plays a key role in reaching therapeutic decisions, thus preventing unnecessary laparotomies. If the decision has been made to observe the patient, repetitive physical evaluations are needed, which also increases accuracy. Therefore, close clinical follow-up is a basic principle of NOM and should be repeated frequently during the hospital stay [3]. An overview of available data on recommendations for clinical examination as well as on other issues discussed below is given in Tables 3 and 4.

**Table 3:** Advice on different practical issues of NOM arising during hospitalisation after blunt hepatic and splenic injuries

	Day 1	Day 2	Day 3	Day 3 → hospital discharge	Comment	References	Highest grade of evidence
Physical and clinical examination	Every 4 h	Every 4 h	Every 4 h	Every 12 h	Recommended for all grades of injuries every 4 h until day 3 and daily until discharge	[3, 29, 30]	2a
Intensity/duration of monitoring	Continuously	Continuously	Continuously	Every 12 h	Recommended for grade I–VI injuries during the first 3 days	[3, 8]	2a
Haemoglobin measurement	Every 6 h	Every 6 h	Every 12 h	Every 24 h	Recommended, every 6–12 h during the first three days, followed by monitoring every 24 h until discharge	[9, 14, 23]	2a
Bed rest	Suggested for grade I–VI lesions	Suggested for grade I–VI lesions	Mobilisation suggested for grade I–VI lesions	Mobilisation suggested for grade I–VI lesions	Mobilisation is recommended between second and third day after injury in haemodynamic stable patients	[32, 33]	2b
Repeat imaging	Not routinely, only in case of deterioration of the patient’s general condition	Ultrasound for grade I–VI lesions	Not routinely	Ultrasound for grade I–VI lesions each second day until discharge	Ultrasound instead of CT scans is suggested in young patients, based on clinical judgment. Routine follow-up CT scanning should not be part of NOM protocols for blunt liver and splenic injuries. Most experts suggest ultrasound monthly after discharge for 6 months (grade I–VI lesions); a single CT scan is recommended 6 months after discharge (lesions grade I–VI)	[34, 35, 36]	2b
DVT prophylaxis	No	No	Yes	Yes	Suggested 48 h after trauma	[37, 38]	2a
Oral intake	No	Yes	Yes	Yes	Allowed 24 h after trauma in haemodynamically stable patients	[29]	2a

*NOM* non-operative management, *DVT* deep vein thrombosis

**Table 4:** Further practical aspects of NOM in blunt hepatic and splenic injuries

	Comment	References	Highest grade of evidence
Transfusion trigger	>2 units of packed red blood cells limit NOM and determine need for OM; conservative treatment may be only continued if transfusion requirements are due to other associated injuries	[23, 39]	2a
Length of stay	Hospital stay is recommended at least for 2 (grade I–II) and 4 (grade III–VI) days, after at least 72 h of admission to a monitored setting, continuous evaluation of vital signs and haemoglobin. Initial admission to ICU is decided upon clinical judgement	[8, 23, 27, 33, 40]	2a
Usual/daily activity	For grade I–III lesions allowed after 2 weeks	[31, 34, 41]	2c
	For grade IV–VI lesions allowed after 6 weeks		
Moderate activity	For grade I–III lesions allowed after 2 months	[31, 34, 41]	2c
	For grade IV–VI lesions allowed after 6 months		
Contact sports	For grade I–III lesions allowed after 6 months	[31, 34, 41]	2c
	For grade IV–VI lesions allowed after 12 months		
Post-splenectomy vaccines against encapsulated bacteria	Immunization is recommended 2–4 weeks after splenectomy	[42, 43]	1a
Post-embolisation vaccines against encapsulated bacteria	Immunization for patients with splenic injuries managed conservatively is not recommended	[44, 45, 46]	2a

*NOM* non-operative management, *OM* operative management, *ICU *intensive care unit

## Intensity and duration of monitoring

Monitoring of vital parameters most commonly includes at least blood pressure, heart rate, **pulse oximetry**

pulse oximetry

and respiratory rate. NOM of hepatic and splenic injuries consists of a period of in-hospital or ICU observation and monitoring. What remains unclear is the duration of necessary observation and the frequency of these interventions [10, 11]. Many clinical studies do not specify the duration of monitoring. In a 10-year review, Raza et al. evaluated vital signs for the first 72 h. In this study, NOM was successful in 89.91% of 1071 patients, where 108 patients showed signs of ongoing **haemorrhage**

haemorrhage,

delayed evidence of hollow viscous perforation or intra-abdominal infection requiring laparotomy [3]. Fernandes et al. developed a rigid protocol for NOM of grade IV splenic injuries. Monitoring consisted of haemoglobin measurement every 6 and arterial blood gas measurement

Experts recommend continuous monitoring of blood pressure, heart rate, oxygen saturation and respiratory rate early after admission

every 12 h on day 1 of admission or even more frequently in the case of clinical deterioration and necessity for ICU admission. NOM failed in 2 patients (7.7%) operated on due to worsening of abdominal pain and **hypovolemic shock**

hypovolemic shock.

No patient developed complications related to the spleen and there were no deaths in this series [8]. According to these findings, many experts recommend a continuous monitoring of at least blood pressure, heart rate and respiratory rate, as well as continuous pulse oximetry during the first days of admission.

## Haemoglobin measurement

Post-injury haemoglobin measurement is another unsolved issue in NOM. Parks et al. reviewed NOM guidelines for blunt abdominal liver injuries. Safety and optimal LOS were evaluated solely based on clinical criteria. They suggested serial haemoglobin measurements every 6 h for the first 24 h in stable patients with grade I–II lesions before discharging patients if they remained stable. In grade III–V injuries they recommended measurements every 6 h during the first 12 h, and after every 12 h thereafter [12]. Gomez et al. reported that 85% of the experts answering a questionnaire concerning haemoglobin monitoring would evaluate values every 8–12 h in NOM of isolated grade II blunt splenic injuries for the time during which the patient is in the ICU [9]. Olthof et al. showed that all experts who participated in their survey perform serial measurement: in the first 24 h every 4–6 h, and every 12–24 h thereafter [23]. Consensus regarding this topic could not be reached until now, but most

Experts suggest haemoglobin controls every 12 h during the first 3 days and every 24 h thereafter

experts suggest close haemoglobin controls at least every 12 h during the first 3 days after trauma, followed by monitoring every 24 h until discharge.

## Number of transfused blood units

The EAST guideline considers that a high number of **blood units**

blood units

transfused early after admission might contraindicate NOM in blunt hepatic or splenic injuries, although there is limited evidence on defined cut-off levels for conversion to surgical treatment [10, 11]. Patients who failed NOM received more blood bags during hospitalization than those who underwent successful conservative treatment. No consensus statement on the number of blood units that contraindicate NOM could be reached [27]. According to Olthof et al., transfusion of five or more units of blood could be a sensible cut-off to guide the decision to operative or perform angiographic intervention [23].

Risk of transfusion-related diseases has led to further discussion and controversy regarding the use of blood products in the treatment of splenic and liver injuries. Luna and Dellinger suggested that the risk of death due to blood transfusion in successful NOM of splenic injury exceeds that of immediate **splenectomy**

splenectomy,

stating thast two blood units in isolated spleen injury limit NOM and conservative treatment may only be continued if transfusion requirements are due to other associated injuries [39]. For

For daily practice, transfusion of more than two blood unit cells limits NOM and determines the need for OM

daily practice, it could be concluded that transfusion of more than two blood unit cells limits NOM and determines the need for operative management (OM).

## Hospital discharge and ICU admission

There are currently no clear recommendations regarding a minimum observation period after blunt splenic and/or liver trauma, neither in the ICU nor on the ward. Recent studies reported that clinical judgment is the predominant aspect for decision-making [40]. In the survey conducted by Olthof et al., all participating experts agreed that the most relevant factors are vital signs and haemoglobin [23]. In a retrospective study performed by Fernandes et al., charts of all patients with splenic injury were reviewed and patients with grade IV lesions treated non-operatively were included in the analysis. LOS was about 5–9 days [8]. Peitzman et al. recommend intensive monitoring for 1 to 3 days, and 3 to 5 days stay on the ward thereafter [27]. A shorter length of stay with successful discharge of patients with low-grade injuries after 1 to 2 days and after 3 to 4 days for higher-grade injuries has also been reported [23]. EAST has not set any recommendations regarding LOS [32]. Additionally, there are no published prospective data about the timing of safe discharge. For clinical practice,

Patients selected for NOM should be monitored continuously

patients selected for NOM should be monitored continuously, some institutional protocols state that all patients with blunt hepatic and splenic injury should be admitted to the ICU [8]. In the survey by Olthof, 100% of the experts administered continuous monitoring, 63% of them in the ICU [23]; 96% agreed to keep monitoring for at least 3 days [33]. To date, clinical judgement still remains the most relevant aspect.

## Bed rest and return to activity

Many surgeons still believe that early mobilisation of patients with blunt solid organ injuries increases the risk of delayed haemorrhage. According to London et al. [34], long periods in bed are unnecessary, since the time of mobilisation after blunt organ injuries does not contribute to late bleeding. In a retrospective cohort study of hepatic injuries, the median day of post-trauma mobilisation was day 2; 30% of patients had been mobilised after the first hospital day, 66% on the second and 80% on the third day. No patients failed NOM as a result of a **secondary bleeding**

secondary bleeding

event from hepatic injuries. Patients with blunt splenic injury were mobilised on median day 3; 17% had been mobilised on the first day, 50% on the second and 77% on the third day. There was no significant difference regarding those who failed NOM due to bleeding upon comparing early versus late mobilisation. Hence, the findings in this retrospective analysis show that the day of first mobilisation was not associated with an increased incidence of delayed rupture. On the contrary, there may be significant benefits of early mobilisation such as reduced hospital LOS and reduced resource utilization [34], as well as lower rates of pneumonia, deep vein **thrombosis**

thrombosis

(DVT) and pulmonary **embolism**

embolism

[41]. Summarising,

Early mobilisation on day 2 or 3 after blunt hepatic or splenic injuries is achievable in clinically stable patients

early mobilisation on day 2 or 3 after blunt hepatic or splenic injuries should be achieved in clinically stable patients.

The EAST guideline does not address the issue of return to activities after hepatic and splenic trauma, probably highlighting the lack of consensus in the literature [10, 11]. Fata et al. reported considerable variation of recommendations for high-grade injuries present among experts. For grade III, IV and V injuries, nearly half of experts would allow return to mild activities within 4 to 6 weeks and to full activities within 2 to 3 months. However, the other half of surveyed participants recommended restricted activities for a period of 4–6 and 5% for more than 6 months [40]. This clearly emphasizes a lack of evidence. Restitution of a simple liver laceration and sub-capsular hematoma occurs within 2 to 4 months, whereas complex injuries require up to 6 months [47]. Some experts recommend return to unrestricted activity only after a normalised CT, usually 3 to 6 months after injury [48]. Most authors relate

The recommended period of reduced activity is related to the severity of injury

the recommendation of reduced activity to the severity of injury. Available published data suggest safety of return to unrestricted activity after splenic trauma between 3 weeks to even 6 months, depending on the severity of the injuries. Clinical judgment represents the key determinant [48].

## Oral intake

The return to normal diet is critical in trauma patients. The guidelines state that issues on early oral feeding are still unsettled in the literature [10, 11]. Gomez et al. mentioned that 71% of experts initiate oral diet in clinically stable patients after 24 h of trauma onset [9].

## Prophylaxis of thromboembolic events

DVT prophylaxis with low-molecular weight **heparin**

heparin

(LMWH) is essential in the management of patients with blunt liver or spleen injuries. In a retrospective analysis conducted in the Regional Trauma Unit of the Sunnybrook Health Science Centre, the largest level 1 trauma facility for adults in Canada, serial impedance **plethysmography**

plethysmography

and lower-extremity contrast **venography**

venography

were performed to detect DVT in a cohort of 716 patients. From January 1989 through April 1991, patients with major trauma were prospectively evaluated with objective diagnostic testing for venous **thromboembolism**

thromboembolism.

DVT in the lower extremities was found in 201 of the 349 patients (58%) without any DVT prophylaxis after trauma. Pulmonary embolism was confirmed in 7 patients [37]. Currently, the optimal DVT prophylaxis strategy for trauma patients with a higher risk of bleeding is unknown. The American College of Chest Physicians recommends

Early initiation of DVT prophylaxis is recommended to reduce the incidence of thromboembolic complications

early initiation of DVT prophylaxis to reduce the incidence of thromboembolic complications [38]. EAST also supports the practice of early DVT prophylaxis for solid organ injuries. However, these recommendations lack a definition of the optimal timing for initiation of DVT prophylaxis [28]. Despite some evidence that pharmacological prophylaxis for DVT does not negatively interfere with NOM, there is no consensus regarding the safest timepoint for its commencement after trauma [10, 11]. In a retrospective study performed by Rostas et al., early use of LMWH during the first 48 h of hospitalization was not associated with bleeding and NOM failure [49]. Another study also suggested that the use of LMWH in the first 48–72 h of admission was not associated with increased blood transfusion rate or NOM failure [42]. Joseph et al. [43] detected no significant correlation between early **anticoagulation**

anticoagulation

and bleeding complications in a North American cohort of patients with blunt abdominal solid organ injuries. Hence, to gain high-quality evidence, prospective studies are required to define the impact of early (within 48 h after trauma) DVT prophylaxis, stratified according to the grade of injury. For clinical practice,

Early prophylaxis of thromboembolic events may be a safe option in trauma patients with blunt injury of a solid organ

early prophylaxis of thromboembolic events may be a safe option in trauma patients with blunt solid organ injury and could be started in the first 48–72 h after trauma.

## Post-splenectomy and post-embolisation vaccination

Overwhelming post-splenectomy infection (OPSI) is a very serious condition that can progress from mild symptoms to fulminant sepsis in a short period of time. Patients who are **asplenic**

asplenic

or **hyposplenic**

hyposplenic

have an increased lifetime risk of OPSI and death, particularly from encapsulated organisms like *Streptococcus pneumonia, Haemophilus influenzae* and *Neisseria meningitidis* [44]. The spleen has specialised lymphoid tissue, including splenic macrophages, that effectively attack these encapsulated organisms. Currently, it is established and recommended that

Vaccines against encapsulated bacteria should be administered 2 weeks before or 2–4 weeks after (elective) splenectomy

vaccines against these bacteria should be administered either 2 weeks before or 2 to 4 weeks after (elective) splenectomy to increase the immunological benefit. Lifelong prophylactic **antibiotics**

antibiotics

should be offered and started in case of any bacterial infection [45]. Routine immunization for patients with splenic injuries managed conservatively without splenectomy is not recommended. Although concerns have been raised about splenic immune function after NOM with or without splenic **angioembolisation**

angioembolisation,

evidence seems to be emerging that immune function is well preserved [46]. A retrospective analysis performed by Tominaga et al. suggested that the immunologic profile of embolised patients is similar to controls [35]. Olthof et al. compared the splenic immune function of patients who were embolised with splenectomised patients and healthy controls. The

Splenic immune function of embolised patients seems to be preserved

splenic immune function of embolised patients was preserved, therefore routine vaccination appears not to be indicated [50].

Both retrospective studies support the safe use of splenic **embolisation**

embolisation

with sustained immune function without a need for vaccination. Concluding, immunisation after splenectomy should be recommended, not, however, after embolisation.

## Repeat imaging

The accuracy of abdominal CT in diagnosing liver trauma excluding other injuries plays a central role in achieving successful NOM. The question of routine follow-up imaging for blunt abdominal trauma has been widely debated. Clancy et al. published that patients with higher-grade injuries were more likely to receive follow-up imaging during their period of hospital admission. CT imaging within 72 h in all grades of splenic injury might be indicated, based on reports documenting the detection of splenic artery **pseudoaneurysm**

pseudoaneurysm

even in low-grade injuries. Factors predicting this pathology are not available. The authors suggest ultrasound instead of CT scans in young patients with grade I and II injuries, based on clinical judgment [51]. CT scans repeated after more than 10 days following initial presentation do not have a substantial influence on patient treatment [36]. The utility of routine follow-up CT scans after blunt hepatic trauma initially managed non-operatively has been evaluated in many studies. Navarro et al. suggested that in asymptomatic paediatric patients, CT follow-up studies do not provide additional information for clinical management guidance. Therefore, routine follow-up imaging studies seem of limited value [48]. Although CT-based documentation of complete organ recovery was once standard of care, follow-up CT is no longer recommended unless clinically indicated [48]. Lynch et al. reported that CT-documented injury was identified by **ultrasound**

ultrasound

for all of the investigated patients; all returned to full activity with no long-term complications after ultrasound demonstrated healing [52]. The main argument for follow-up imaging is delayed splenic rupture. Radiographic healing may under- or overestimate physiologic healing [48]. Therefore, routine radiological follow-up of blunt splenic injury does not necessarily identify patients at risk nor does it help to guide a recommendation for return to full activity after an appropriate time of restriction. In another retrospective study by Allins et al., none of the follow-up scans showed major progression of injury. The authors therefore concluded that these scans did not influence decision-making for a change to surgery in any of the patients and are therefore unnecessary. Hence, routine follow-up CT scanning should not be part of NOM protocols for blunt liver and splenic injuries [53].

## Practical conclusion

Hepatic and splenic injuries are common and NOM shows increasing popularity although good evidence, i.e., from well-designed prospective trials, is lacking. Especially questions relating to daily life (return to activity, follow-up, bed rest, LMWH treatment) remain controversial. Developing protocols and regular auditing might be the first steps to successfully achieving better outcomes and avoiding unnecessary surgery with all its long-term consequences. Nevertheless, surgery at the right time is still the right choice to save a patient’s life. The use of standardized operating procedures (SOPs) to drive NOM of blunt hepatic and splenic injuries is uncommon in many centres, leading to a high number of different management pathways, even among surgeons working in the same hospital. SOPs could help to triage appropriate patients for either continuous monitoring or therapeutic intervention. There is emerging evidence that the use of strict protocols for NOM of hepatic and splenic injuries might increase overall success rates, thus avoiding unnecessary operations.

## DFP-Literaturstudium

Blunt abdominal trauma of liver and spleen: which answer is correct?

Blunt abdominal trauma is not very frequent in central European emergency departments.

Abdominal organs are involved in 10% of polytrauma patients, with an occurrence of hepatic and splenic injuries in 5 and 5%, respectively.

The only possible therapeutic strategy is surgical treatment.

Due to frequent postoperative complications after primary surgical treatment in the past, a paradigm shift to non-operative management (NOM) in haemodynamically stable patients has emerged in major trauma centres.

Regarding therapeutic laparotomies, evidence has shown additional advantages such as less frequent need for blood transfusions, lower mortality rates and lower healthcare costs compared with conservative treatment

The Moore scoring system is considered the gold standard to describe hepatic and splenic injuries: which answer is correct?

This classification scheme is an anatomic description scaled from I to VI for the spleen and from I to VIII for the liver, representing minimal to the most severe injury.

Most liver injuries are grade IV or V and are successfully treated conservatively.

Laceration involving segmental or hilar vessels producing major devascularisation (>25% of spleen) in splenic injuries is described as Moore grade IV.

For multiple injuries, two grades should be advanced in the classification system, up to grade III.

The Moore score is based on the Organ Injury Scale (OIS) of the American Association for Surgery of Trauma (AAST) and was published in 2000.

Bed rest and return to activity after blunt hepatic and splenic injuries: which answer is correct?

Many surgeons still believe that early mobilisation of patients with blunt solid organ injuries increases the risk of delayed haemorrhage.

The guidelines report clear recommendations concerning the issue of return to activities after hepatic and splenic trauma.

Restitution of a simple liver laceration and sub-capsular hematoma occurs within 8 to 10 months, whereas complex injuries require up to 12 months.

For grade III, IV and V injuries, nearly half of experts would allow return to full activities within 2 to 3 weeks.

Some experts recommend return to unrestricted activity only after a normalised computer tomography (CT), usually 1 to 3 months after injury.

Post-splenectomy and post-embolisation vaccination: which answer is correct?

Overwhelming post-splenectomy infection (OPSI) is a mild condition that rarely causes complications like fulminant sepsis.

Patients who are asplenic or hyposplenic have an increased lifetime risk of OPSI and death especially from encapsulated organisms like *Pseudomonas aeruginosa* or *Escherichia coli.*

Currently, it is established and recommended that vaccines against these bacteria should be administered either 4 weeks before or 8 to 10 weeks after (elective) splenectomy.

Routine immunization for patients with splenic injuries managed conservatively without splenectomy is also recommended.

Retrospective studies analysed the splenic immune function of embolised patients and concluded that routine vaccination appears not indicated, because splenic immune function was preserved.

Repeat imaging: which answer is correct?

CT scans repeated after more than 2 weeks following initial presentation have a substantial influence on patient treatment.

Many authors suggest ultrasound instead of CT scans in young patients with grade I and II splenic injuries.

Routine follow-up CT scanning is considered an integrated part of NOM protocols for blunt liver and splenic injuries.

In asymptomatic paediatric patients, CT follow-up studies always provide additional information for clinical management guidance.

Routine radiological follow-up of blunt splenic injury always helps to guide a recommendation for return to full activity.
